# Cellular mechanism for herbal medicine Junchoto to facilitate intestinal Cl^−^/water secretion that involves cAMP-dependent activation of CFTR

**DOI:** 10.1007/s11418-018-1207-9

**Published:** 2018-03-22

**Authors:** Tomohiro Numata, Kaori Sato-Numata, Yasunobu Okada, Ryuji Inoue

**Affiliations:** 1 Department of Physiology, Graduate School of Medical Sciences, Fukuoka University, 7-45-1 Nanakuma, Johnan-ku, Fukuoka, 814-0180 Japan; 2 Japan Society for the Promotion of Science, Chiyoda-ku, Tokyo, Japan; 3 Department of Molecular Cell Physiology Graduate School of Medical Science, Kyoto Prefectural University of Medicine, Kamigyo-ku, Kyoto, 602-8566 Japan

**Keywords:** Junchoto, CFTR, Herbal medicine, Intestinal fluid, Kampo

## Abstract

Constipation is a common symptom frequently compromising the quality of daily life. Several mechanistically different drugs have been used to mitigate constipation, including Japanese herbal (Kampo) medicines. However, the mechanisms of their actions are often not well understood. Here we aimed to investigate the molecular mechanisms underlying the effects of Junchoto (JCT), a Kampo medicine empirically prescribed for chronic constipation. Cl^−^ channel activity was measured by the patch-clamp method in human cystic fibrosis transmembrane conductance regulator (CFTR)-expressing HEK293T cells and human intestinal Caco-2 cells. cAMP was measured by a luciferase-based assay. Cell volume change was measured by a particle-sizing and particle-counting analyzer and video-microscopic measurement. In both CFTR-expressing HEK293T and Caco-2 cells, JCT dose-dependently induced whole-cell currents showing typical biophysical and pharmacological features of CFTR. Robust expression of CFTR was confirmed by RT-PCR and Western blotting in Caco-2 cells. Luciferase-based measurement revealed that JCT increases intracellular cAMP levels. Administration of the adenylate cyclase inhibitor SQ22536 or CFTR inhibitor-172, or treatment with small interfering RNAs (siRNA) targeting CFTR, abolished JCT-induced whole-cell currents, suggesting that elevated intracellular cAMP by JCT causes activation of CFTR in Caco-2 cells. Finally, blockade of CFTR activity by CFTR inhibitor-172 or siRNA-knockdown of CFTR or application of SQ22536 markedly reduced the degree of cell volume decrease induced by JCT. JCT can induce a Cl^−^ efflux through the CFTR channel to promote water secretion, and this effect is likely mediated by increased cAMP production.

## Introduction

Constipation affects multiple aspects of a person’s health, including health-related quality of life. It is one of the most frequently reported functional gastrointestinal disorders. The prevalence of constipation varies from 2.6 to 26.9%, being most frequent in females and in advanced age [1, 2]. Constipation is caused by decreased internal organ function, lack of water fluid, etc. [3]. In general, improvement in dietary habits, water intake, exercise and other activities in daily life is prioritized for relieving constipation, but more active interventions become indispensable for severe constipation. In recent years, a novel chloride-channel activator, lubiprostone, which is not classified as a prokinetic, has been developed [4] and is attracting attention. The mechanism of lubiprostone’s laxative actions is accounted for by the activation of Chloride Channel-2 (ClC-2) channels that results in chloride efflux across the apical membrane and subsequent paracellular passive movement of sodium and water into the intestinal lumen. The luminal distension caused by increased intestinal fluid then promotes gut motility, thereby enhancing intestinal and colonic transits [4]. However, there is contrasting evidence that the molecular target of lubiprostone may rather be the cystic fibrosis transmembrane conductance regulator (CFTR) channel than ClC-2. In the *Xenopus* oocyte expression system, CFTR but not ClC-2 has been found to be activated via the prostaglandin receptor sub-type 4 (EP-4) [5]. In the intestinal epithelia of both mice and human, endogenous expression of CFTR is restricted to the apical membrane while that of ClC-2 is localized largely in the basolateral membrane, and, moreover, only the former can be activated by lubiprostone [6]. Thus, it still remains controversial what type of ion channels/transporters are involved in lubiprostone’s laxative actions. It is also reported that guanylate cyclase-C (GC-C) receptor activators, linaclotide and plecanatide, exert similar gastrokinetic actions, through enhanced intracellular cGMP synthesis and subsequent phosphorylation of CFTR protein by cGMP-dependent protein kinase II (PKG II), which facilitates luminal chloride secretion and paracellular movement of sodium and water [3, 7].

Kampo medicines are composed of various medicinal herbs. Two classes of Kampo medicines, Rhei Rhizoma-based (class 1) and Kenchuto-based ones (class 2) are frequently used for the treatment of constipation [8]. In Rhei Rhizoma-based medicines, Junchoto (JCT) and Mashiningan (MNG) constitute a unique subgroup that contains Cannabis Fructus, as well as a small amount of Rhei Rhizoma. JCT and MNG are prescribed exclusively for elderly patients suffering from spastic constipation, which results mostly in softened stool. Recently, it was suggested that such laxative actions of JCT and MNG may involve CFTR activation [9, 10]. However, this speculation relies entirely on the presumptive specificity of an organic CFTR inhibitor used (CFTRinh-172) which also inhibits other types of Cl^−^ channels including volume-sensitive anion channels [11] and ClC-2 [12] at micromolar concentrations, thus lacking rigorous proof at the molecular level.

In the present study, we therefore adopted more direct gene-based approaches to manipulate CFTR expression, in order to unequivocally determine the molecular target of JCT’s actions. Furthermore, to confirm whether JCT can actually promote water secretion as the consequence of CFTR activation (or induction of Cl^−^ efflux), we compared the time courses of and causal relationship between JCT-induced cell volume decrease and CFTR activation. Additionally, the cellular mechanism by which JCT induces CFTR-mediated Cl^−^ conductance was investigated in some detail.

## Methods

### Reagents

DMSO was purchased from Wako Pure Chemical Industries Ltd. (Osaka, Japan). Forskolin, CFTR inhibitor-172 and SQ22536 were obtained from Sigma-Aldrich (St. Louis, MO, USA). KT5823 was obtained from Cayman (Cayman Chemical Co, Ann Arbor, MI, USA). Junchoto compound was obtained from Tsumura (Tsumura Co., Ltd, Tokyo, Japan: http://www.tsumura.co.jp/english/products/pi/JPR_T051.pdf). Junchoto powder was dissolved in DMSO at concentrations from 400 to 800 mg/mL and used on the same day. All other chemical reagents were purchased from commercial suppliers.

### Cell cultures and cDNA expression

HEK293T cells and Caco-2 cells were grown in Dulbecco’s modified Eagle’s medium (DMEM) supplemented with 10% fetal bovine serum, 30 units/ml penicillin and 30 μg/ml streptomycin (in the case of Caco-2 cells, 1% non-essential amino acids were further added), under a 95% air–5% CO_2_ atmosphere at 37 °C. Twenty-four hours after plating, HEK293T cells were transfected with either pCIneo-IRES-GFP vector or human CFTR-pCIneo-IRES-GFP vector (a generous gift from Dr. RZ Sabirov [13]). Lipofectamine 2000 (Invitrogen, Carlsbad, CA, USA) was used as a transfection reagent following the manufacturer’s instructions. Electrophysiological measurements and Western blot analysis were performed 36–72 h after transfection.

### Mean cell volume measurements

Mean cell volume was measured at room temperature by electronic sizing with a Coulter-type cell size analyzer (CDA-500; Sysmex, Hyogo, Japan). The mean volume of the cell population was calculated from the cell volume distribution measured after the machine was calibrated with latex beads of known volume. Isotonic “Tyrode solution” (300 mosmol/kg H_2_O adjusted by d-mannitol) contained (in mM) 140 NaCl, 5 KCl, 1 MgCl_2_, 2 CaCl_2_, 10 d-glucose and 10 HEPES (pH 7.4 adjusted by NaOH). Relative cell volumes in Fig. 6a–d are defined by the following equation: relative cell volume = *V*_A_/*V*_Ctl_, where *V*_Ctl_ and *V*_A_ are the mean cell volumes before and after DMSO (control) or JCT application, respectively.

### Single-cell size measurements

Single-cell size was measured at room temperature in cells adhering to a non-coated cover glass in Tyrode solution. The experiments were performed in a 1-ml recording chamber in which the cover glass was placed. The cells were visualized through a charge-coupled device camera (XC-ST70, Sony, Tokyo, Japan) and images were recorded with the mAgicTV software (I-O DATA, Ishikawa, Japan). The cross-sectional area (CSA) of the cell of interest was measured as an indicator of cell size by ImageJ software [14]. Relative CSAs in Fig. 6e and h are defined by the following equation: relative CSA = *A*_A_/*A*_Ctl_, where *A*_Ctl_ and *A*_A_ are the CSA values before and after JCT application, respectively.

### Electrophysiology

After transfection with human CFTR-pCIneo-IRES-GFP or pCIneo-IRES-GFP plasmid, cells were dissociated by mechanical agitation and lodged onto coverslips placed in tissue culture dishes. Membrane currents of these cells were recorded at room temperature (22–27 °C) using the whole-cell mode of the patch-clamp technique, with an Axopatch 200B (Axon Instruments/Molecular Devices, Union City, CA, USA) patch-clamp amplifier. For whole-cell recordings, patch electrodes prepared from borosilicate glass capillaries had an input resistance of 3–5 MΩ. Current signals were filtered at 5 kHz with a four-pole Bessel filter and digitized at 20 kHz. pCLAMP (version 10.5.1.0; Axon Instruments/Molecular Devices) software was used for command pulse control, data acquisition and analysis. Data were also analyzed using Origin (OriginLab Corp., Northampton, MA, USA) software. For whole-cell recordings, the series resistance was compensated (to 70–80%) to minimize voltage errors. The external solution contained (in mM) 110 CsCl, 2 CaCl_2_, 1 MgCl_2_, 5 glucose and 10 HEPES (pH 7.4 adjusted with CsOH, and osmolality adjusted to 310 mmol/kg with d-mannitol). The pipette solution contained (in mM) 110 CsCl, 2 MgSO_4_, 1 EGTA, 10 HEPES, 1 Na_2_ATP and 15 Na-HEPES (pH 7.4 adjusted with CsOH, and osmolality adjusted to 300 mmol/kg with d-mannitol). To test the ion selectivity of the macroscopic channel currents, 110 mM Cs-aspartate in the bath solution was replaced with 55 mM CsCl, 2 mM CaCl_2_, 1 mM MgCl_2_, 5 mM glucose and 10 mM HEPES (pH 7.4 adjusted with CsOH, and osmolality adjusted to 310 mmol/kg with d-mannitol). To test the nystatin-perforated whole-cell currents with single-cell size measurements, the Na^+^-based bath solution contained (in mM) 140 NaCl, 5 KCl, 2 CaCl_2_, 1 MgCl_2_, 10 HEPES and 10 d-glucose (pH adjusted to 7.4 with NaOH, and osmolality adjusted to 320 mosmol/kgH_2_O with d-mannitol). The pipette solution contained (in mM) 55 K_2_SO_4_, 20 KCl, 5 MgCl_2_, 0.2 EGTA and 5 HEPES (pH adjusted to 7.4 with KOH, and osmolality adjusted to 300 mosmol/kgH_2_O with d-mannitol).

### Western blot analysis

After 36 h of transfection, Caco-2 cells were solubilized in the radioimmunoprecipitation assay (RIPA) buffer (pH 8.0) containing 0.1% SDS, 0.5% sodium deoxycholate, 1% Nonidet P40, 150 mM NaCl, 50 mM Tris–HCl, 1 mM PMSF and 10 μg/μl leupeptin, then centrifuged at 17,400*g* for 20 min. Whole-cell lysates were fractionated by 7.5% SDS-PAGE and electro-transferred onto a poly-vinylidene fluoride (PVDF) membrane. The blots were incubated with anti-CFTR antibody (1:1000 dilution, CUSABIO and CUSAb, MD, USA: CSB-PA001608) or monoclonal anti-α-tubulin (as an internal standard, 1:2000 dilution; Sigma-Aldrich: T6074), and stained using the enhanced chemiluminescence system (Thermo Scientific, Rockford, IL, USA).

### RNA isolation and RT-PCR

Total cellular RNA was extracted from Caco-2 cells by using NucleoSpin^®^ RNA Plus (Takara-Bio, Shiga, Japan) according to the protocol supplied by the manufacturer. The concentration and purity of RNA were determined using a Nanodrop-ND1000 (Thermo Fisher Scientific, Waltham, MA, USA). Total RNA samples were reverse-transcribed at 42 °C for 30 min with Prime Script RTase using the PrimeScript™ II High Fidelity RT-PCR Kit (Takara-Bio, Shiga, Japan), according to the manufacturer’s protocols. Expression levels of CFTR in the cDNA from Caco-2 were determined by PCR. As a positive control, we amplified the partial sequence of glyceraldehyde-3-phosphate dehydrogenase (GAPDH). Suppression of RNA expression was confirmed by RT-PCR analysis. PCR was done using KOD-Plus-Ver.2 (Toyobo, Osaka, Japan) under the following conditions: pre-denaturation at 94 °C for 2 min, followed by 32–35 cycles of denaturation at 98 °C for 10 s and annealing at 55–63 °C for 30 s, and final extension at 68 °C for 30 s. The sequences of gene-specific primers (synthesized by Sigma-Aldrich) and the predicted lengths of PCR products are as follows: hGAPDH (496 bp) forward and reverse primers: 5′-GGTGAAGGTCGGAGTCAACG-3′ and 5′-CAAAGTTGTCATGGATGACC-3′ respectively; hCFTR (327 bp) forward and reverse primers: 5′-AGGAGGAACGCTCTATCG-3′ and 5′-GCAGACGCCTGTAACAAC-3′, respectively.

### siRNA transfection

Caco-2 cells were transfected with 1 µg small interfering RNA (siRNA) using the RNAiMAX Reagent (Thermo Fisher Scientific) following the manufacturer’s instructions, and used for experiments 48–72 h later. To determine transfection efficacy, we used the pEGFP-N1 vector (Takara-Bio). As a negative control, we used a non-silencing siRNA (or mock siRNA). The mock siRNA and the siRNA against CFTR were purchased from Bioneer (Bioneer Daejon, S. Korea).

### Statistical evaluation

All data are expressed as mean ± SEM. We accumulated the data for each condition from at least three independent experiments. Statistical analyses were performed using Student’s *t* test. *P* < 0.05 was considered significant.

## Results

### Concentration-dependent activation of CFTR-mediated Cl^−^ current by Junchoto

**Fig. 1:**
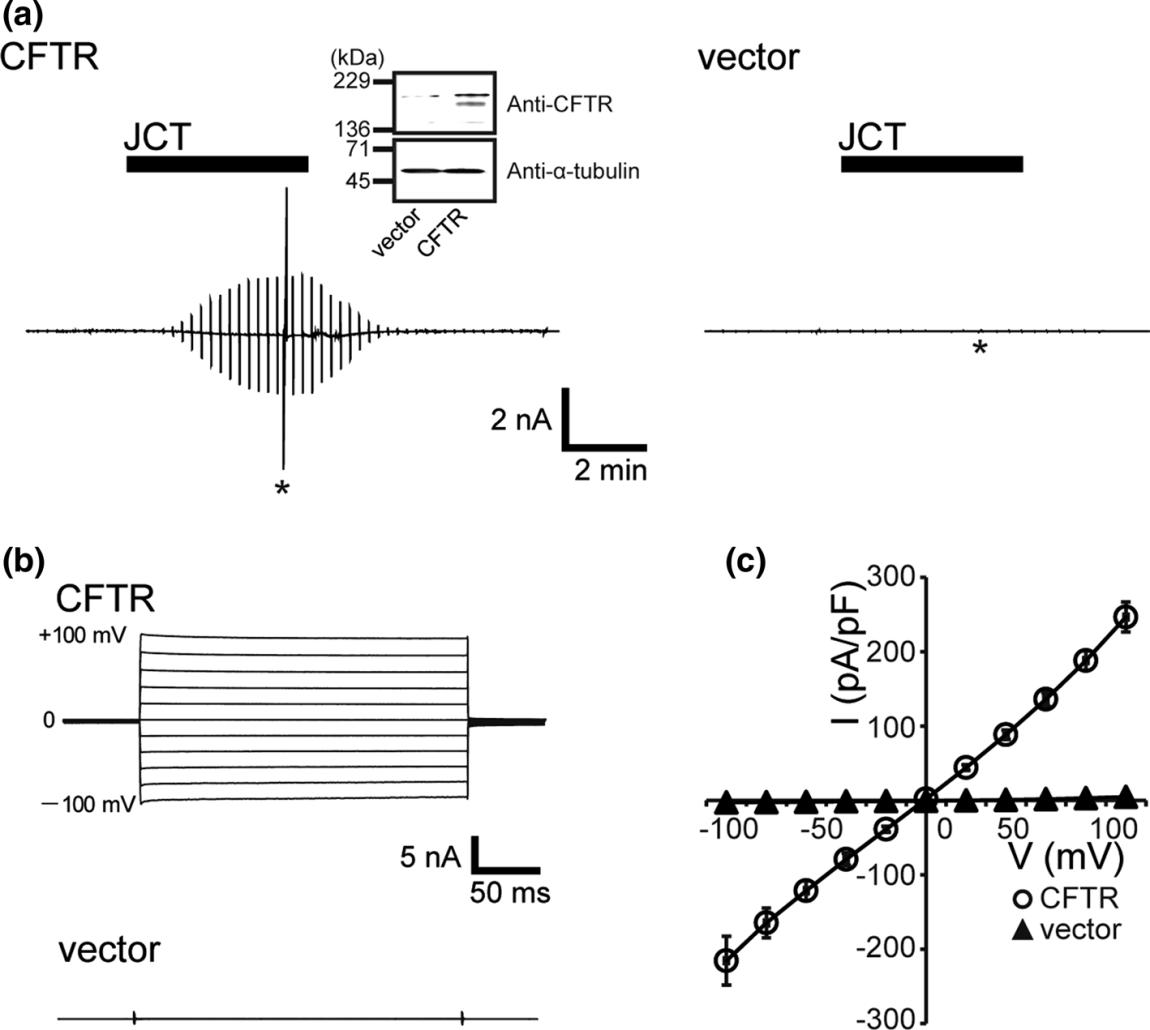
Junchoto (JCT)-induced CFTR currents in HEK293T cells transiently transfected with CFTR. **a** Representative records of whole-cell current activation in CFTR- (*left*) and vector-transfected (*right*) cells before and after application of 400 μg/mL JCT (*filled bars*), taken during the application of alternating pulses from 0 to ± 40 mV every 10 s. The *asterisks* denote times when step pulses were applied. The *inset* shows a membrane displaying immunoblot of CFTR protein from control (vector-transfected) and CFTR transfected HEK293T cells in the upper lane. Note that the lower band is only detected in CFTR-transfected HEK293T cells. Alpha-tubulin bands with molecular mass 50 kDa were detected at equal levels in the lower lane. **b** The current response to step pulses from −100 to +100 mV for CFTR (*top traces*) and the vector (*bottom trace*). **c** *I*–*V* relationships for the mean JCT-activated current densities for cells expressing CFTR (*open circles*; *n* = 6) and vector (*filled triangles*; *n* = 6)

Previous studies with short-circuit measurements using the Ussing chamber reported that Junchoto (JCT) increased the net membrane current across a polarized human bronchial epithelial cell layer [9]. Since this trans-epithelial current was effectively reduced by the CFTR inhibitor-172 [9], it was simply concluded that the current arose from CFTR activation. However, the electrophysiological details of this current remain entirely undetermined. To more unequivocally investigate the identification of JCT’s target, we first performed patch-clamp measurements in HEK293T cells over-expressing human CFTR channels (Fig. 1a, inset). As shown in Fig. 1a (left panel), application of JCT resulted in slow development of a large whole-cell current. No such current was induced in empty vector-transfected cells (Fig. 1a, right trace: Fig. 1b, bottom traces). The JCT-induced current responded to step voltage pulses with almost instantaneous activation and deactivation time courses (Fig. 1b, upper traces), and showed a linear current–voltage (*I*–*V*) relationship (Fig. 1c, open circles). These electrophysiological properties are characteristic of heterologously expressed CFTR-mediated Cl^−^ channels, suggesting that JCT is a robust activator of the CFTR channel.

**Fig. 2:**
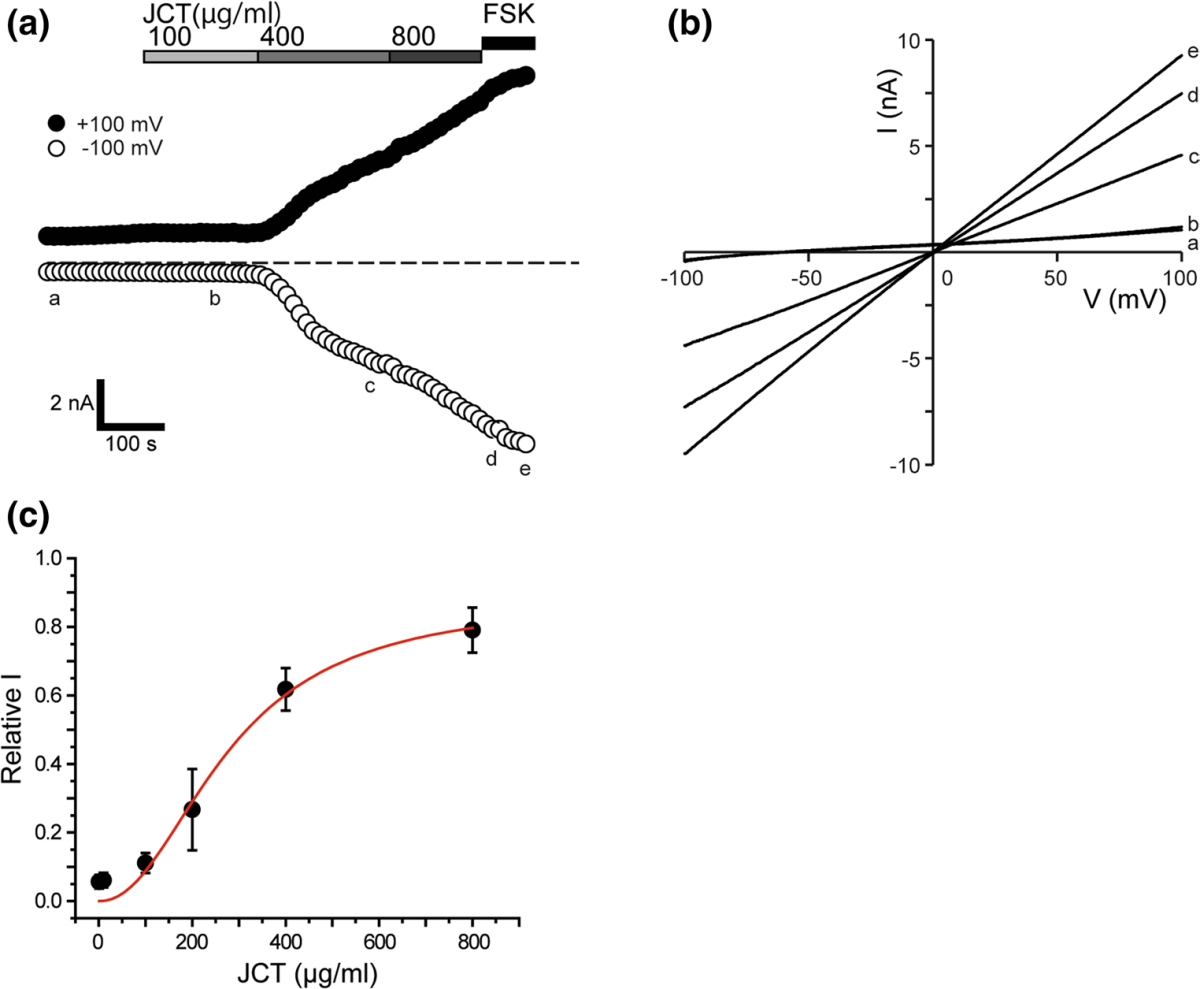
JCT dose–response of CFTR channel in HEK293T cells transiently transfected with CFTR. **a** Representative time courses of the JCT evoked whole-cell currents recorded at +100 and −100 mV under ramp clamp. **b** Corresponding *I*–*V* relationships at time points a, b, c, d and e. **c** Peak current densities induced by JCT normalized to that of 10 μM FSK (*n* = 5–8). Data points show the mean ± S.E.M

Figure 2a demonstrates the concentration-dependent effects of JCT on inducing Cl^−^ current assessed by a cumulative application protocol. To attain maximal activation, forskolin (10 μM) was applied at the end of the protocol. The degree of activation of Cl^−^ current by JCT (expressed as the fraction of forskolin-induced Cl^−^ current) was increased in a concentration-dependent manner, and half maximal activation (at +100 mV) occurred at 279 µg/ml (EC_50_) with a cooperativity coefficient of 2.14 (Fig. 2c).

### Junchoto activates endogenous CFTR channels in Caco-2 cells

**Fig. 3:**
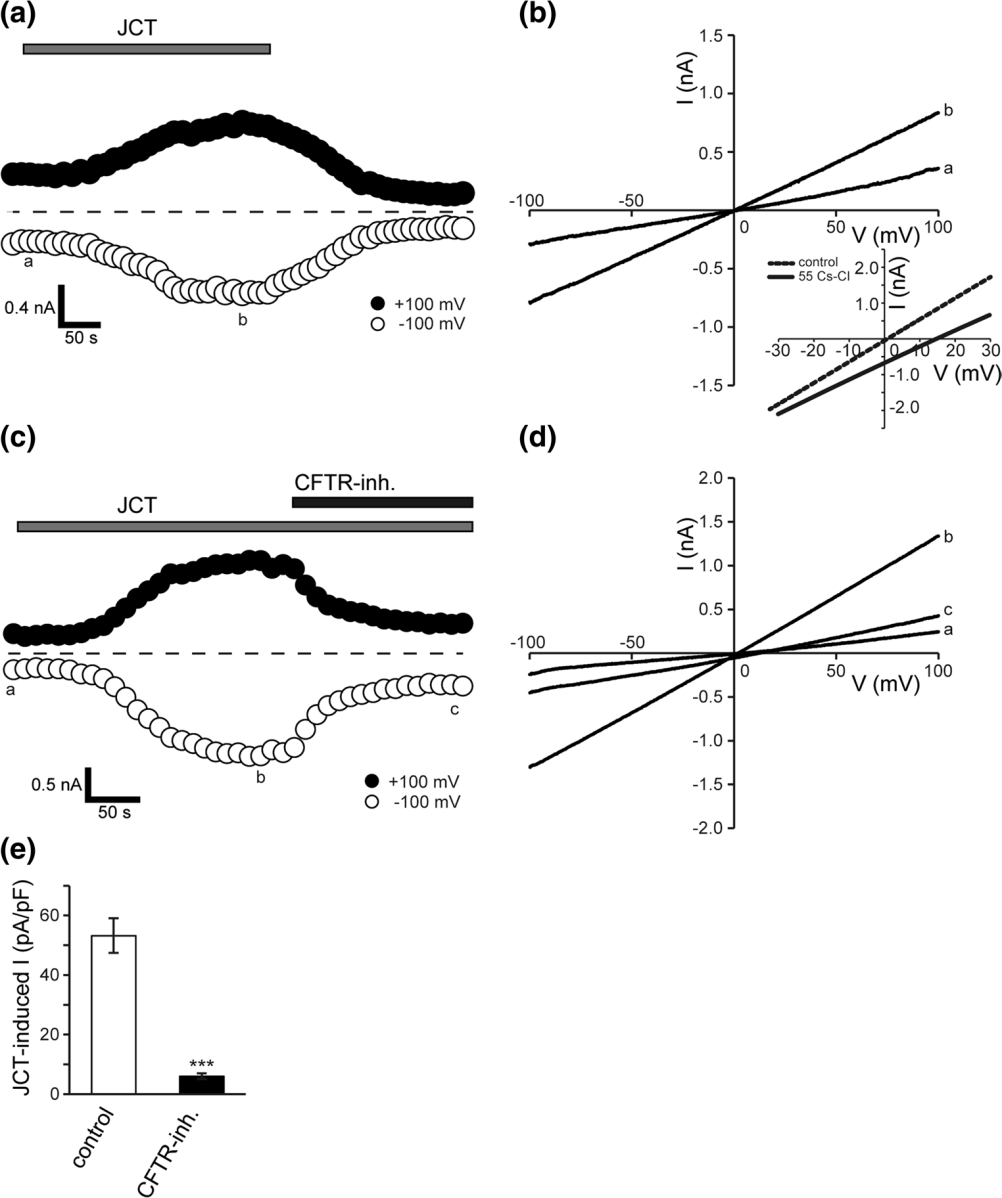
Whole-cell currents evoked by JCT in human colonic Caco-2 cells. **a**, **c** Representative time courses of the JCT-evoked whole-cell currents recorded at +100 and −100 mV under ramp clamp. *Gray bar* and *solid bar* show application of 400 μg/mL of JCT and 20 μM of CFTR inhibitor-172 (*CFTR-inh*.), respectively. **b**, **d** Corresponding *I*–*V* relationships at time points a, b and c. **e** Averages of JCT-induced whole-cell current in control and CFTR inh. (*n* = 5–6). The *inset* shows JCT-induced whole-cell current when the extracellular Cl^−^ concentration is reduced from control to 55 mM CsCl (*55 Cs-Cl*) bath solution. Data points show the mean ± SEM. ***P < 0.001 compared to control at +100 mV

In the next step, to test the effects of JCT on endogenous CFTR channels, we repeated similar experiments in Caco-2 cells. As in CFTR-expressing HEK293T cells, JCT induced whole-cell Cl^−^ currents that developed slowly and had a linear *I*–*V* relationship with a reversal potential near 0 mV under symmetric Cl^−^ conditions (Fig. 3a, b). When the extracellular Cl^−^ concentration was reduced from 116 to 61 mM (from 110 to 55 mM CsCl bath solution), the reversal potential of JCT-activated current shifted to the right (Fig. 3b: inset) by 14.1 ± 0.8 mV (*n* = 6). This value is in good agreement with that predicted for an ideal anion electrode (15.1 mV). JCT-induced currents were blocked by extracellular application of the so-called CFTR specific inhibitor, CFTR-inhibitor-172 (CFTR-inh.: Fig. 3c–e). These properties are essentially the same as those of recombinant CFTR channels observed elsewhere.

### Suppression of JCT-induced currents by siRNA of CFTR

**Fig. 4:**
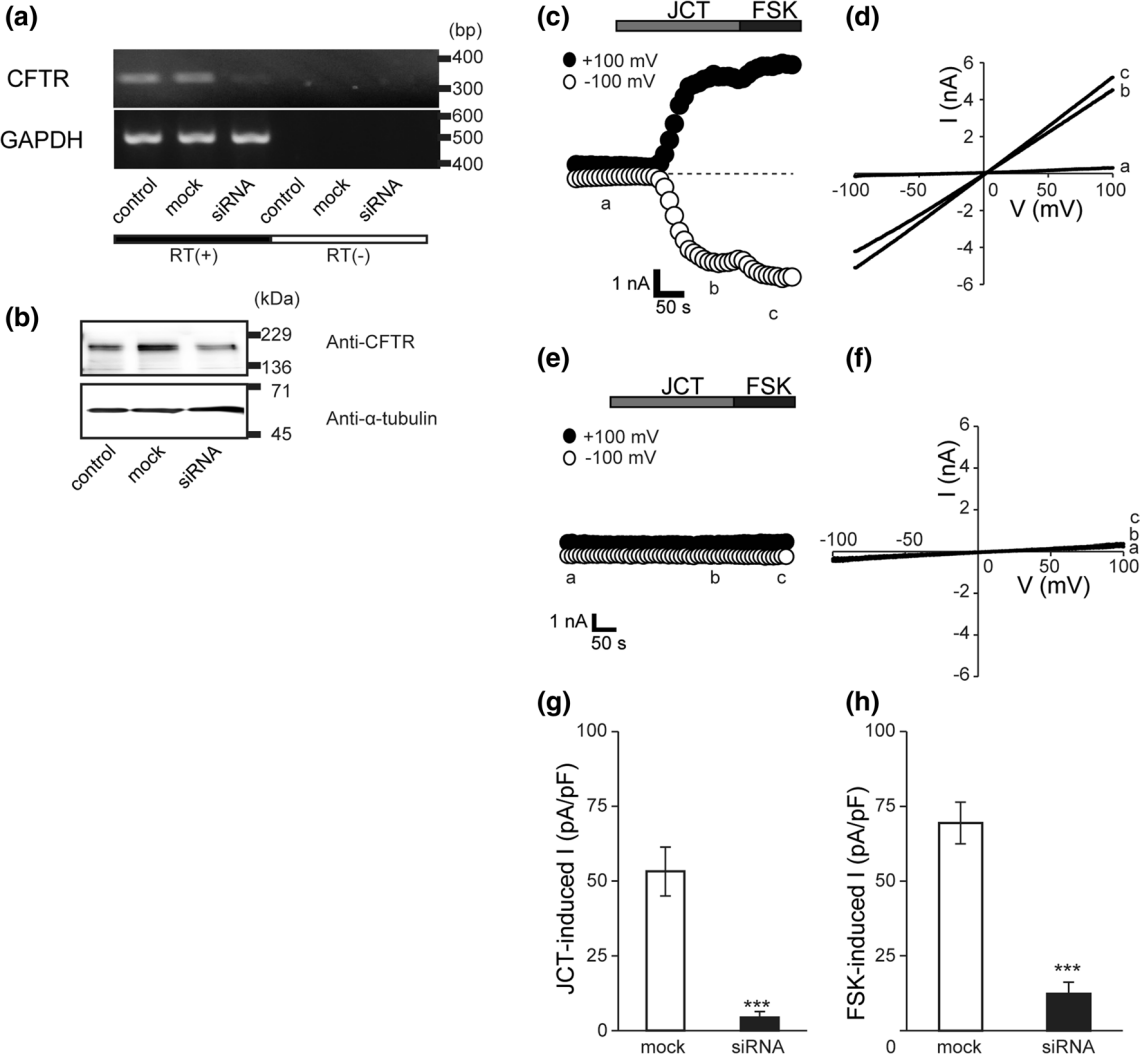
Effects of small interfering RNA (siRNA) silencing of CFTR on JCT-induced anion currents in Caco-2 cells. **a** RT-PCR analysis of CFTR mRNA and glyceraldehyde-3-phosphate dehydrogenase (GAPDH) mRNA in control, mock-treated and CFTR siRNA-treated Caco-2 cells. The data represent 3 similar experiments. No PCR product was amplified when reverse transcriptase was omitted from the reaction in the RT(−) group. The nucleotide sequences of the PCR products obtained with CFTR-specific primers were completely identical to the corresponding sequences in human CFTR (4302-4722 Sequence ID: NM_000492.3). **b** Immunoblot of CFTR protein from control, mock-treated and CFTR siRNA-treated Caco-2 cells. Alpha-tubulin bands with molecular mass 50 kDa were detected at equal levels. **c**–**h** Representative time courses of the JCT-evoked whole-cell currents recorded at +100 and −100 mV under ramp clamp in mock siRNA-treated cells (**c**) and in CFTR siRNA-treated cells (**e**). *Gray bar* and *solid bar* show application of 400 μg/mL of JCT and 10 μM of FSK, respectively. Corresponding *I*–*V* relationships at time points a–c in **d** and a–c in **f**. **g**, **h** Averages of JCT-induced whole-cell current at +100 mV in mock and CFTR siRNA (**g**), and FSK-induced whole-cell current (**h**) (*n* = 5–6). Data points show the mean ± SEM. *P < 0.05 compared to mock at +100 mV

To confirm that the JCT-induced current in Caco-2 cells indeed reflects the activation of the CFTR channel, we next conducted an RT-PCR assay to detect the *cftr* mRNA expression. As shown in Fig. 4a (top left), robust amplification of the *cftr* transcript of an expected size (327 bp) occurred from the reverse-transcribed RNA of Caco-2 cells. Moreover, 24-h treatment of Caco-2 cells with CFTR-specific siRNA almost completely eliminated the *cftr* transcript, whereas mock siRNA had no effect. Neither siRNA affected the mRNA expression of a housekeeping enzyme GAPDH (Fig. 4a, top right).

Western blot analyses were performed for whole-cell lysate extracted from Caco-2 cells treated with none (control), mock siRNA or CFTR-siRNA with a polyclonal anti-CFTR antibody (Fig. 4b). The immunoreactive bands detected in control and mock-transfection lanes had a molecular mass of 168 kDa, and this band was diminished by CFTR-siRNA treatment.

The functional impact of siRNA was confirmed by patch-clamp experiments. While JCT failed to induce noticeable membrane currents after CFTR-specific siRNA transfection (Fig. 4e–g), it still induced robust currents in mock siRNA-transfected Caco-2 cells (Fig. 4c, d, g). Essentially the same results were obtained when FSK, instead of JCT, was used as a stimulant (Fig. 4c–h).

These results collectively suggest that endogenous CFTR protein of Caco-2 cells is functionally and effectively activated by JCT.

### Mechanisms of CFTR channel activation

It is well known that the CFTR channel is regulated by cyclic nucleotides such as cAMP and cGMP. Because of its apical localization in intestinal epithelia, this channel serves as the main route for Cl^−^ and HCO_3_^−^ effluxes to the lumen [15]. Therefore, to investigate the cellular mechanism underlying CFTR activation by JCT in Caco-2 cells, we employed KT5823 and SQ22536, relatively specific inhibitors for protein kinase G (PKG) and adenylate cyclase (AC), respectively.

**Fig. 5:**
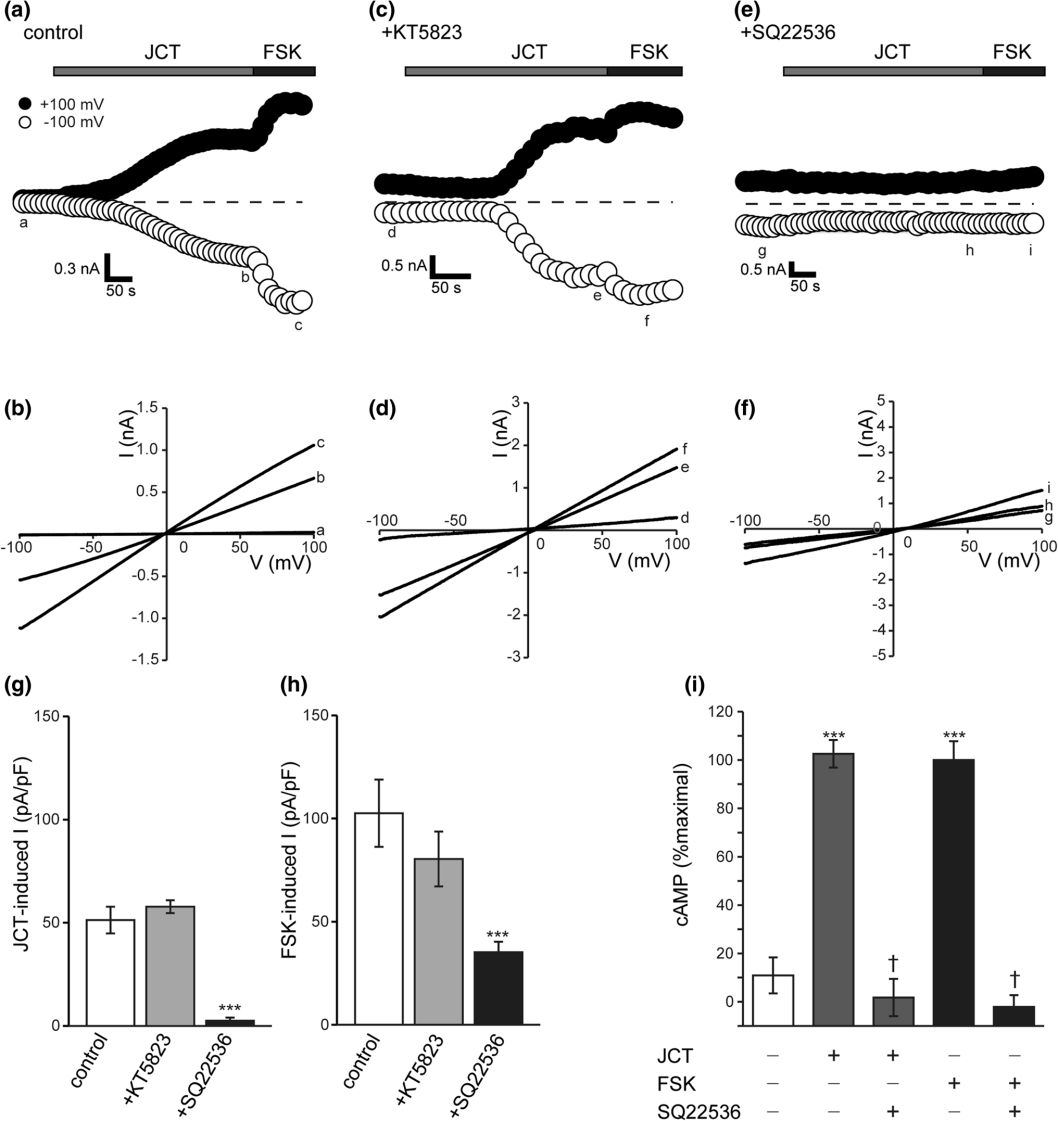
JCT-induced CFTR activation is mediated by adenylyl cyclase and cAMP signaling pathway. Effects of KT5823 (1 μM) and SQ22536 (100 μM) were measured on whole-cell currents activated by JCT (400 μg/ml) in Caco-2 cells. Representative time courses and corresponding *I*–*V* relationships in control (**a**, **b**), KT5823 (**c**, **d**) and SQ22536 (**e**, **f**). Current densities evoked by JCT (**g**) and FSK (10 μM) (**h**) (*n* = 5–6). Averages of JCT-induced whole-cell current densities recorded at +100 mV (**g**), and FSK-induced whole-cell current densities (**h**) (*n* = 5–6). Data points are the mean ± SEM. ***P < 0.001. **i** Biochemical determination of cAMP levels. Caco-2 cells were pretreated with or without SQ22536 (100 μM), and then treated with JCT (400 μg/ml) or FSK (10 μM). Data are expressed as a percentage of the cAMP levels (*n* = 6 independent experiments). ***P < 0.001 compared with control, ^†^*P* < 0.001 cAMP levels in cells pretreated with JCT or FSK. *Error bars* indicate the SEM

As demonstrated and summarized in Fig. 5, the magnitude of JCT-induced current was not significantly affected by KT5823 treatment (Fig. 5a–d, g). In contrast, SQ22536 almost completely eliminated JCT-induced currents (Fig. 5e–g), suggesting that cAMP rather than cGMP mediates CFTR channel activation by JCT. Essentially the same results were obtained with FSK (Fig. 5a–f, h). To reinforce these observations further, we next assessed the ability of JCT to stimulate intracellular cAMP synthesis using a luciferase-based chemiluminescence method. As anticipated, JCT elicited an increase in chemiluminescence, which was antagonized by pretreatment with SQ22536 at a concentration which effectively inhibited FSK-induced cAMP increase (Fig. 5i).

### Evaluation of epithelial fluid secretion by cell volume measurements

Solute secretion from the cell inevitably accompanies osmotic water movement to cause cell volume decrease. We therefore estimated the extent of water secretion caused by JCT-induced Cl^−^ efflux via CFTR in Caco-2 cells by using a Coulter-type volume measurement and video-microscopic measurement of secretory volume decrease (SVD) [16].

**Fig. 6:**
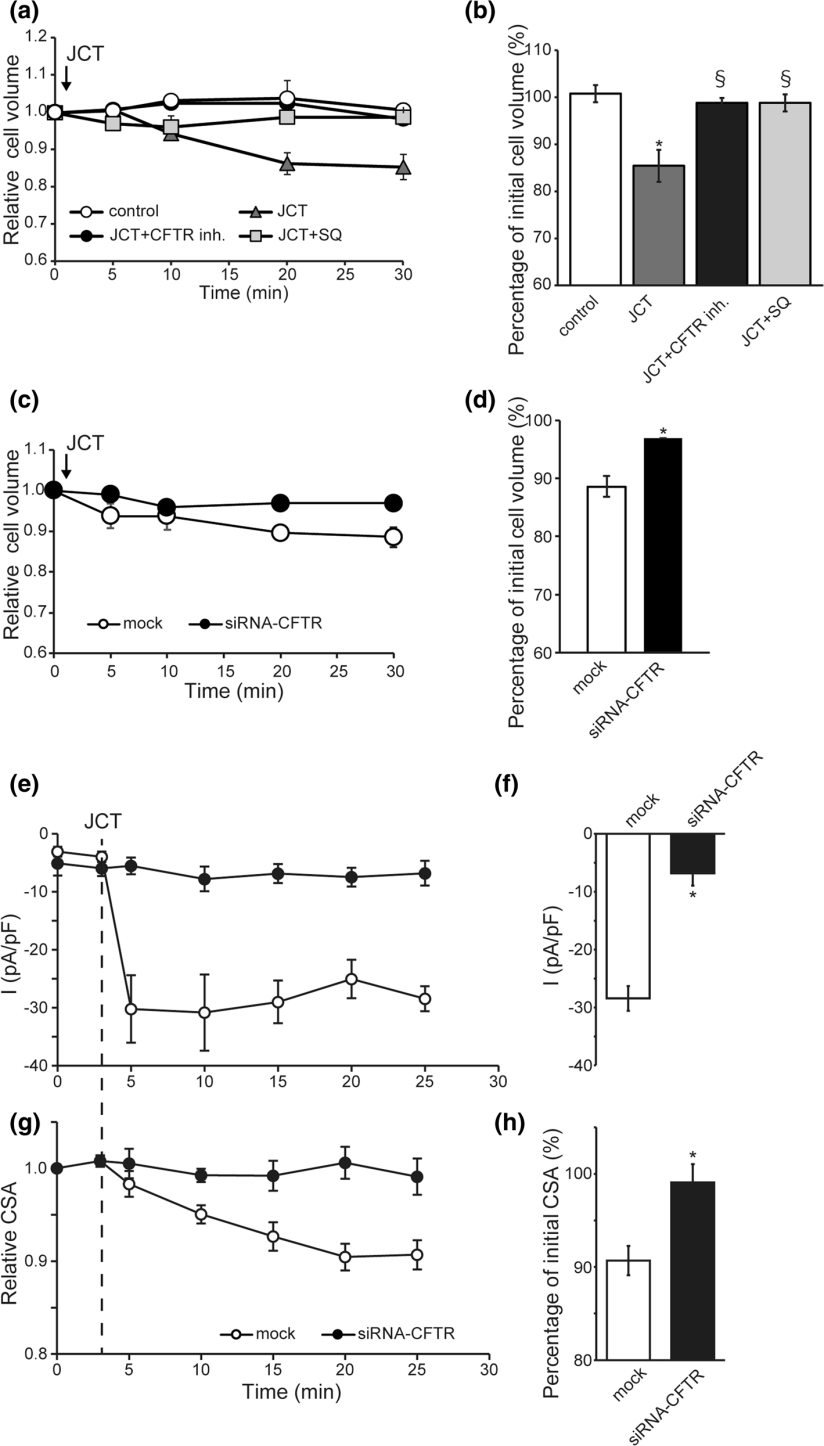
Involvement of the CFTR channel in JCT-induced cell-volume decrease of Caco-2 cells. **a** Time course of changes in mean cell volume. At time 0, JCT (400 μg/ml) was applied except in control. The conditions denote JCT, JCT with SQ22536 (100 μM), and JCT with CFTR-inh. (20 μM) on secretory volume decrease (SVD) monitored by an electronic sizing technique. **b** Percentage of initial cell volume (SVD) at 30 min (*n* = 5–10). **c** Effects of treatment with CFTR siRNA or mock siRNA on SVD. **d** Percentage of initial cell volume (SVD) at 30 min after application with or without JCT (*n* = 5). Data points are the mean ± SEM. *P < 0.05 compared to control or mock. ^§^*P *< 0.05 compared to JCT. **e–f** Time course of the JCT-induced inward current and cell volume decrease. **e** Averaged time courses of the JCT-evoked whole-cell currents recorded at −100 mV under ramp clamp in mock siRNA-treated cells and in CFTR siRNA-treated cells with nystatin-perforated recording. The holding potential was at −60 mV. **f** Averages of JCT-induced whole cell current at −100 mV (*n* = 6–7). Data points are the mean ± SEM. *P < 0.05. **g** Time-dependent profile of JCT-induced cell volume change in mock siRNA-treated cells and in CFTR siRNA-treated cells. JCT (400 μg/ml) was applied at 3 min. **h** Percentage of initial CSA at 22 min after application with JCT (*n* = 6–7). Data points are the mean ± SEM. *P < 0.05 compared to mock

As shown in Fig. 6a and b, JCT applied to non-treated Caco-2 cells (control) caused a ~15% SVD at 30 min after its administration. The extent of SVD was significantly diminished when CFTR channel activity was blocked by CFTR inhibitor-172 (CFTR-inh.: 20 μM) or SQ22536 (SQ: 100 μM). More prominent SVD (~24%) occurred when CFTR-mediated Cl^−^ efflux was maximally activated by FSK (10 μM) (data not shown). The extent of SVD of Caco-2 cells was also greatly reduced by CFTR-specific siRNA treatment, while mock siRNA had little effect (Fig. 6c, d). Simultaneous measurement of whole-cell currents and cross-sectional area (CSA) was performed by combining the patch-clamp technique and video-microscopy. Under nystatin-perforated whole-cell recording, administration of 400 µg/mL JCT into the bath evoked an inward current at a holding potential of −60 mV (Fig. 6e). The whole-cell current reached a peak within a few minutes and persisted. The JCT-induced current was almost completely blocked by CFTR-specific siRNA (Fig. 6e, f). Administration of JCT reduced the size of Caco-2 cells and its time course paralleled the development of the inward current (Fig. 6e). This cell volume decrease was greatly attenuated by CFTR-siRNA treatment (Fig. 6g, h).

It is thus highly likely that cAMP-dependent activation of the CFTR channel significantly contributes to the JCT-induced SVD of Caco-2 cells, which involves facilitated Cl^−^ efflux and accompanying water movement.

## Discussion and conclusions

The results of the present study make it apparent that JCT can facilitate Cl^−^ and water secretion via cAMP-dependent activation of the CFTR channel. Several lines of evidence strongly support this view. First, JCT dose-dependently induced Cl^−^ currents showing the hallmark properties of CFTR channels in both a heterologous expression system (HEK293T cells) and a cultured intestinal epithelial cell line (Caco-2 cells). Second, the JCT-induced current in Caco-2 cells was not only suppressed pharmacologically (i.e. by CFTR inhibitor-172) but also effectively eliminated by specific siRNA knockdown of CFTR. Third, the activation of CFTR-mediated Cl^−^ current in Caco-2 cells by JCT was eliminated by the adenylate cyclase inhibitor SQ22536 (but not the PKG inhibitor KT5823), thus likely involving a cAMP-dependent mechanism. Finally, the JCT-induced secretory volume decrease of Caco-2 cells was greatly attenuated by CFTR inhibitor-172, CFTR-specific siRNA and SQ22536 with similar efficacies to inhibit CFTR-mediated Cl^−^ currents. This observation is interpreted as indicating that facilitated Cl^−^ efflux through the CFTR channel by JCT promoted water secretion from Caco-2 cells and caused their volume size reduction.

The possibility that JCT may activate epithelial CFTR was previously suggested by short-circuit current measurements in human bronchial epithelia [9]. However, this was based merely on the inhibitory effect of a relatively specific CFTR inhibitor, CFTR inhibitor-172, at a concentration (20 μM) which non-specifically inhibited other types of Cl^−^ channels [11, 12]. In this regard, the main progress of the present study lies in the unequivocal demonstration that JCT activates CFTR and facilitates subsequent water secretion, as well as clarification of the mechanism for this drug’s actions (i.e. via stimulated cAMP synthesis).

The functional significance of CFTR-mediated Cl^−^ secretion has been implicated in various epithelia, including the proximal small intestine, by the use of CFTR knockout mice. For instance, the ablation of the *cftr* gene is found to cause intestinal obstruction due to decreased epithelial fluid secretion [17, 18]. Mutations of this gene in human cystic fibrosis patients are also well known to accompany severe secretory defects in various organs such as exocrine glands, airways and the gastrointestinal tract [19], which frequently culminates in luminal obstruction. Thus, there is little doubt that targeting CFTR, which resides predominantly in the apical surface of epithelia, would be rational and beneficial for alleviating the symptoms associated with defective epithelial secretion such as constipation.

Forskolin is an established adenylate cyclase activator that is made from the root of a plant in the mint family (*Coleus forskohlii*) and is capable of inducing the maximal activity of CFTR at 10 μM [20]. In our experiments as well, this concentration of forskolin maximally activated the CFTR channel (Fig. 2) as well as markedly increasing the intracellular cAMP level (Fig. 5). Importantly, the estimated efficacy of JCT (at 800 μg/ml) in the present study is comparable to that of forskolin (10 μM) in both activating the recombinant CFTR channel and stimulating cAMP synthesis in Caco-2 cells (Figs. 2 and 5). The potency of JCT to activate CFTR (half-maximal activation occurs at 279 μg/ml) is also similar to that to induce significant cell volume decrease or Cl^−^/water secretion in an intestinal epithelial cell line Caco-2 (Fig. 6). These pharmacological profiles strongly point to the clinical utility of this Kampo compound for re-hydrating dry contents in the intestine. Indeed, the evidence supportive of these observations has been briefly described: oral administration of JCT (300 or 1000 mg/kg) greatly improves opioid-induced severe constipation in a rat model with increased fecal count and dried fecal weight [9]. A similar CFTR-targeted improvement of constipation has been investigated in detail in a rat model treated with another Kampo medicine, Mashiningan, although in this case stimulation of cGMP-mediated signaling was presumed to be responsible for CFTR activation [10]. However, again, whether CFTR is involved here was not unequivocally proved because of the considerably higher concentration of the drug used to selectively inhibit CFTR (CFTR-inhbitor-172, 20 μM).

The active ingredient(s) of JCT which stimulate cAMP production and thereby cause CFTR activation/water movement remains to be determined. Junchoto consists of 10 crude plant-extract herbs (viz. *Cannabis Fructus, Aurantii Fructus Immaturus, Rhei Rhizoma, Magnoliae Cortex, Paeoniae Radixm, Glycyrrhizae radix, Rehmanniae Radix, Angelicae Radix, Scutellariae Radix, Persicae Semen*) (http://wakankensaku.inm.u-toyama.ac.jp/wiki/Main_Page), each of which contains many active ingredients. Consultation with the literature shows that, of the 10 herbs, only three (*Paeoniae Radixm, Glycyrrhizae radix, Scutellariae Radix*) may have cAMP-mediated actions. Paeoniflorin, an active ingredient from *Paeoniae Radixm*, was shown to stimulate noradrenaline release from nerve terminals in a Ca^2+^- and cAMP-dependent manner, probably through an as-yet-unelucidated mechanism involving tetrodotoxin-sensitive presynaptic depolarization [21]. GU-7, a 3-arylcoumarin derivative extracted from *Glycyrrhizae radix*, was found capable of increasing the intraplatelet cAMP concentration to inhibit platelet aggregation through phosphodiesterase (PDE) inhibition [22]. A more recent study using isoform-specific inhibitors has revealed that this compound (GU-7 or glycycoumarin) can dose-dependently accumulate intracellular cAMP (but not cGMP) via specific inhibition of type-3 PDE activity [23]. Finally, the most striking finding is that biacalein, a major flavonoid extracted from *Scutellariae Radix*, likely stimulates Cl^−^ secretion across rat colon epithelia. More detailed investigations using human colonal epithelial T84 cells suggested that baicalein causes a dose-dependent increase in a short-circuit current representing the apical Cl^−^ efflux via enhanced cAMP production without affecting the intracellular Ca^2+^ level [24]. These multiple actions through both stimulating the synthesis and inhibiting the degradation of cAMP would render Junchoto a potent cAMP-producing agent and thus an effective CFTR activator whose efficacy is comparable to that of forskolin (Figs. 2, 5). Interestingly, another mechanistically similar laxative, Mashiningan, does not contain two of the above three active ingredients (viz. glycycoumarin, baicalein). This difference may distinguish the laxative efficacies of Junchoto and Mashiningan. Indeed, the former is generally believed to be superior to the latter in softening stools. This powerful re-hydrating action of Junchoto may also be beneficial for secretory disorders in other organs such as airways, pancreas, salivary glands [25], eyes [26] (e.g. dry eyes) and uterus/oviduct [27] (e.g. infertility).

In addition, it is becoming widely recognized that beside their well-known anti-oxidant effects, flavonoids modulate many biological functions by opening K^+^ channels, blocking voltage-dependent Ca^2+^ channels, decreasing inflammatory signals, modulating apoptotic processes [28] and activating/inhibiting epithelial Cl^−^ transports via, e.g., CFTR channels (stimulation; biacalein, tangeretin; inhibition: quercetin, lutelin) [29, 30]. These last effects potentially are expected to underlie the development of new anti-diarrheals and laxatives based on the ‘flavonoid’ pharmacophore [31].
